# Lithium suppresses Aβ pathology by inhibiting translation in an adult *Drosophila* model of Alzheimer's disease

**DOI:** 10.3389/fnagi.2014.00190

**Published:** 2014-07-30

**Authors:** Oyinkan Sofola-Adesakin, Jorge I. Castillo-Quan, Charalampos Rallis, Luke S. Tain, Ivana Bjedov, Iain Rogers, Li Li, Pedro Martinez, Mobina Khericha, Melissa Cabecinha, Jürg Bähler, Linda Partridge

**Affiliations:** ^1^Department of Genetics, Evolution and Environment, Institute of Healthy Ageing, University College LondonLondon, UK; ^2^Max Planck Institute for Biology of AgeingCologne, Germany; ^3^Laboratory of Molecular Biology of Cancer, UCL Cancer InstituteLondon, UK

**Keywords:** lithium, Drosophila, Alzheimer's disease, translation, lifespan

## Abstract

The greatest risk factor for Alzheimer's disease (AD) is age, and changes in the ageing nervous system are likely contributors to AD pathology. Amyloid beta (Aβ) accumulation, which occurs as a result of the amyloidogenic processing of amyloid precursor protein (APP), is thought to initiate the pathogenesis of AD, eventually leading to neuronal cell death. Previously, we developed an adult-onset *Drosophila* model of AD. Mutant Aβ42 accumulation led to increased mortality and neuronal dysfunction in the adult flies. Furthermore, we showed that lithium reduced Aβ42 protein, but not mRNA, and was able to rescue Aβ42-induced toxicity. In the current study, we investigated the mechanism/s by which lithium modulates Aβ42 protein levels and Aβ42 induced toxicity in the fly model. We found that lithium caused a reduction in protein synthesis in *Drosophila* and hence the level of Aβ42. At both the low and high doses tested, lithium rescued the locomotory defects induced by Aβ42, but it rescued lifespan only at lower doses, suggesting that long-term, high-dose lithium treatment may have induced toxicity. Lithium also down-regulated translation in the fission yeast *Schizosaccharomyces pombe* associated with increased chronological lifespan. Our data highlight a role for lithium and reduced protein synthesis as potential therapeutic targets for AD pathogenesis.

## Introduction

Alzheimer's disease (AD) is the most common form of dementia in the ageing population. The proportion of deaths due to heart disease and stroke decreased by 13 and 20% respectively between 2000 and 2008, while those due to AD increased by a staggering 66% (Alzheimer's Association, 2012). AD is a neurodegenerative disorder characterized by the presence of amyloid beta (Aβ) deposits and neurofibrillary, hyperphosphorylated tau tangles in the brain (Spires and Hyman, 2005). Age is the major risk factor for AD, and the fruit fly *Drosophila* has been used to demonstrate experimentally that the neurons of older adult flies are intrinsically more susceptible to Aβ toxicity (Rogers et al., 2012). The ageing process could contribute to increased vulnerability to protein toxicity through several routes, including reduced protein turnover through inefficient proteasome- and autophagy-mediated clearance mechanisms (Rubinsztein et al., 2011; Rogers et al., 2012). Interestingly, Aβ accumulation has been linked to several processes affected by ageing. For instance, in a *Drosophila* model of AD, Aβ increased the appearance of abnormal autophagic vesicles, which lost their structural integrity and function with age, and thus influenced neuronal integrity (Ling et al., 2009).

In a previous study, we developed an adult-onset *Drosophila* model of AD, using an inducible gene expression system to express Arctic mutant Aβ42 specifically in adult neurons (Sofola et al., 2010). Aβ42 accumulated in these flies and they displayed increased mortality together with progressive neuronal dysfunction. We also demonstrated that, if we treated the adult flies expressing Aβ chronically with lithium, we rescued toxicity caused by Aβ. Furthermore, we found that Aβ protein, but not mRNA levels were reduced upon lithium treatment (Sofola et al., 2010).

Lithium has been used to treat psychiatric conditions such as bipolar disorder, and it also has interesting neuroprotective effects (Rybakowski, 2011). Lithium is able to promote neurogenesis, and increase the levels of neurotrophins such as brain-derived neurotrophic factor (BDNF), and to inhibit glycogen synthase kinase-3 (GSK-3), which is involved in AD (Machado-Vieira et al., 2009; Rybakowski, 2011). Lithium also reduces amyloid production by affecting APP processing/cleavage in cells and mice, presumably by down regulating the levels of phosphorylated APP (Phiel et al., 2003; Rockenstein et al., 2007).

Lithium can also influence various ageing-regulated processes that could interfere with protein turnover and consequently affect neurological function. For instance, lithium has been shown to induce autophagy (Sarkar et al., 2005), promote proteasome-mediated degradation (Jing et al., 2013), and influence components of the translational machinery (Bosetti et al., 2002; Karyo et al., 2010). Also, inhibiting GSK-3 in HCC1806 cells by a GSK-3 inhibitor or knockdown of GSK-3β has been reported to significantly decrease polysome assembly, and thus affect translation (Shin et al., 2014). GSK-3 was shown in these cells to partially exert its effects on translation via eIF4E-binding protein 1 (4E-BP1). Knocking down 4E-BP1 only partially restored the cap-dependent translation suppressed by GSK-3 inhibition, suggesting that GSK-3β may regulate other components involved in protein synthesis (Shin et al., 2014).

In this study, we investigated underlying mechanism/s by which lithium can reduce Aβ protein levels and thus pathology in the adult-onset, *Drosophila* model of AD. Aβ peptide is directly expressed in this model, and therefore, any effect of lithium on Aβ levels is not due to its ability to alter APP processing, but rather a consequence of its role in protein synthesis or degradation.

## Materials and methods

### Fly stocks and maintenance

All fly stocks were maintained at 25°C on a 12:12-h light:dark cycle at constant humidity on a standard sugar-yeast (SY) medium (15 gl^−1^ agar, 50 gl^−1^ sugar, 100 gl^−1^ autolyzed yeast, 100 gl^−1^ nipagin and 3 ml l^−1^ propionic acid). Adult-onset, neuronal-specific expression of Arctic mutant Aβ42 peptide was achieved by using the elav GeneSwitch (elavGS)-UAS system [GAL4-dependent upstream activator sequence; (Osterwalder et al., 2001)]. ElavGS was derived from the original elavGS 301.2 line (Osterwalder et al., 2001) and obtained as a generous gift from Dr H. Tricoire (CNRS, France). UAS-ArcAβ42 were obtained from Crowther et al. (2005). elavGS and UAS-lines used in all experiments were backcrossed six times into the *w*^1118^ genetic background. For the fly AD model, flies carrying homozygous UAS-ArcAβ42;elavGS constructs were out-crossed to either w1118 flies, or flies expressing EGFP in a w1118 background; and adult-onset neuronal expression was induced in the female progeny by treatment with mifepristone (RU486; 200 μM) added to the standard SY medium.

### Lithium treatment protocol

Lithium chloride solution was made at 10 M concentration and added to 200 μM RU486 standard SY medium for final concentrations of lithium.

### Lifespan analyses

For all experiments, flies were raised at a standard density on standard SY medium in 200 mL bottles. Two days after eclosion once-mated females were transferred to experimental vials containing SY medium with or without RU486 (200 μM) at a density of 15 flies per vial. Deaths were scored almost every other day and flies were transferred to fresh food. Data are presented as survival curves and statistical analysis was performed using log-rank tests to compare survival of groups.

### Negative geotaxis assays

Climbing assays were performed at 25°C according to previously published methods (Sofola et al., 2010). Climbing was analyzed every 2–3 days post-RU486 treatment. Fifteen adult flies were placed in a vertical column (25 cm long, 1.5 cm diameter) with a conic bottom end, tapped to the bottom of the column, then their climb to the top of the column was analyzed. Flies reaching the top and flies remaining at the bottom of the column after a 45 s period were counted separately, and three trials were performed at 1 min intervals for each experiment. Scores recorded were the mean number of flies at the top (*n*_top_), the mean number of flies at the bottom (*n*_bottom_) and the total number of flies assessed (*n*_tot_). A performance index (PI) defined as ½(*n*_tot_ + *n*_top_ - *n*_bottom_)/ *n*_tot_) was calculated. Data are presented as the mean PI ± s.e.m. obtained in three independent experiments for each group, and analyses of variances (ANOVA) were performed using JMP 10.0 software.

### Quantification of Aβ42 peptide

To extract total Aβ42, five *Drosophila* heads were homogenized in 50 μl GnHCl extraction buffer (5 M Guanidine HCl, 50 mM Hepes pH 7.3, protease inhibitor cocktail (Sigma, P8340) and 5 mM EDTA), centrifuged at 21,000 g for 5 min at 4°C, and cleared supernatant retained as the total fly Aβ42 sample. Aβ42 content was measured in Arctic mutant Aβ42 flies and controls using the hAmyloid β42 ELISA kit (HS) (Millipore). Samples were diluted in sample/standard dilution buffer and the ELISA performed according to the manufacturers' instructions. Protein extracts were quantified using the Bradford protein assay (Bio-Rad protein assay reagent; Bio-Rad laboratories (UK) Ltd) and the amount of Aβ42 in each sample expressed as a ratio of the total protein content (pmol/g total protein). Data are expressed as the mean ± s.e.m. obtained in three independent experiments for each genotype. ANOVAs and Tukey's-HSD *post-hoc* analyses were performed using JMP 7.0 software.

### Western blotting

The same number *of Drosophila* heads for each sample were homogenized in Laemmli sample buffer containing β-mercaptoethanol and boiled for 10 min. Proteins were separated on SDS polyacrylamide gels and blotted onto nitrocellulose membranes. Membranes were incubated in a blocking solution containing 5% milk proteins in TBST for 1 hr at room temperature, then probed with primary antibodies diluted in TBST + 5% BSA overnight at 4°C. Antibodies were from Cell Signaling unless specified. GFP antibody was used at 1 in 1000 dilution (2955), phospho eIF2B and total eIF2B at 1 in 1000 (Ab4775 and Ab32713), phospho and total eEF2 at 1 in 500 (2331 and 2332), and phospho S6K **(**9206), total S6K at 1 in 1000 [made from previously published sequence (Pearson et al., 1995)]. Appropriate secondary antibodies were used at a dilution of 1 in 10,000.

### Proteasome activity

Fly heads were homogenized in 25 mM Tris, pH 7.5 and protein content determined by Bradford assay. Chymotrypsin-like peptidase activity of the proteasome was assayed the using fluorogenic peptide substrate Succinyl-Leu-Leu-Val-Tyr-amidomethylcoumarin (LLVY-AMC), based on a previously published protocol (Bulteau et al., 2002; Rogers et al., 2012). 20 μg of crude fly head homogenate total protein was incubated at 37°C with 25 μM LLVY-AMC in a final volume of 200 μLs. Enzymatic kinetics were measured in a temperature-controlled microplate fluorimeter (Tecan Infinite M200), at excitation/emission wavelengths of 360/460 nm, measuring fluorescence every 2 min for 1 h. Proteasome activity was determined as the slope of AMC accumulation over time per mg of total protein (pmoles/min/mg).

### ^35^S-methionine incorporation

Protein translation was measured in fly heads using a method adapted from Bjedov et al. (2010). Standard SY medium was first supplemented with 100 μCi ^35^S methionine/mL of food (American Radiolabeled Chemicals 1mCi/37MBq ARS0104A). 15 flies were transferred to each vial containing 1 mL radioactive SY medium. After three-hours of feeding, flies were transferred to non-radioactive SY for 30 min in order to purge any undigested radioactive food from the intestines. Flies that were in contact with the radioactive food for 1 min were used as a background control. Flies were then decapitated using liquid nitrogen and the heads and bodies homogenized in 1% SDS and heated for 5 min at 95°C. Samples were centrifuged twice for 5 min at 16,000 g. Proteins were precipitated by the addition of the same volume of 20% cold TCA (10% TCA final concentration) and incubated for 15 min on ice. Samples were then centrifuged at 16,000 g for 15 min, the pellet washed twice with acetone and re-suspended in 200 μl of 4 M guanidine-HCl.

Samples (100 μl) were mixed with 3 mL of Fluoran-Safe 2, BDH and radioactivity counted in a liquid scintillation analyzer (TriCarb 2800TR, Perkin Elmer), with appropriate quench corrections. SDS-homogenates, prior to TCA precipitation, were also sampled and analyzed as a measure of the total radioactivity (incorporated and un-incorporated) present. Total protein for each sample was determined by Bradford assay and a translation index was calculated as follows: (TCA protein cpm/total cpm)/μg protein per sample.

### Chronological lifespan assays in fission yeast

Standard *S. pombe* laboratory strain *972 h^−^* cells were grown in EMM2 as previously described (Rallis et al., 2013). When cultures reached a stable maximal density, cells were harvested, serially diluted and plated on YES plates. The measurement of Colony Forming Units (CFUs) was taken as time-point 0 at the beginning of CLS curve (i.e., 100% cell survival). Measurements of CFUs were conducted on the following days. Error bars represent standard deviation calculated from three independent cultures, with each culture measured three times at each time-point. Statistical significance (*t*-test) was determined at the time-point when medial lifespan (50% cells dead) was reached for the untreated cells.

### Polysome profiling in fission yeast

Translational profiles were acquired as previously described (Rallis et al., 2013). Briefly, *S. pombe* cells were treated with 100 μM Cycloheximide for 5 min. Cells were then collected by centrifugation at 3500 rpm for 5 min and diluted in 20 mM Tris-HCl pH 7.5, 50 mM KCL, 10 mM MgCl supplemented with protease (PMSF), 100 μM cycloheximide, 1 mM DTT and 200 ng/mL Heparin. Cells were lysed in a Fastprep-24 machine using glass beads. Sucrose gradients (10–50%) were generated using a Biocomp Gradient Master, and protein preparations were loaded and centrifuged at 35,000 rpm for 2 h 40 min. Polysome gradients were then loaded to the fractionator to obtain the translational profiles.

### Polysome profiling in *drosophila*

Polysome profiles were generated as previously described with minor modifications (Dinkova et al., 2005). Heads (300) were homogenized on ice in 1200 μl polysome extraction buffer (300 mM NaCl, 50 mM Tris-HCL (pH 8.0), 10 mM MgCl_2_, 1 mM EGTA, 200 mg heparin/ml, 400 U RNAsin/ml, 1.0 mM phenylmethylsulfonyl fluoride, 0.2 mg cycloheximide/ml, 1% Triton X-100, 0.1% Sodium Deoxycholate). Lysates were mixed gently and placed on ice for 10 min. Debris was the removed by spinning at 20,000 g (4°C) for 10 min and the supernatant was layered onto a 10–50% sucrose gradient in high salt resolving buffer (140 mM NaCl, 25 mM Tris-HCL (pH 8.0), 10 mM MgCl_2_). Using a Beckman SW41Ti rotor (38,000 rpm at 90 min, 4°C) polysomes and ribosomal subunits were separated before the gradients were fractionated. Profiles were continuously monitored (Ab 252 nm) using a Teledyne density gradient fractionator.

### Statistical analyses

Data are presented as means ± s.e.m. obtained in at least three independent experiments. JMP (version 10.0) software (SAS Institute, Cary, NC, USA) was used for data analyses.

## Results

### Lithium reduced Aβ load in arctic Aβ42 expressing flies through protein clearance/degradation-independent mechanisms

**Figure 1:**
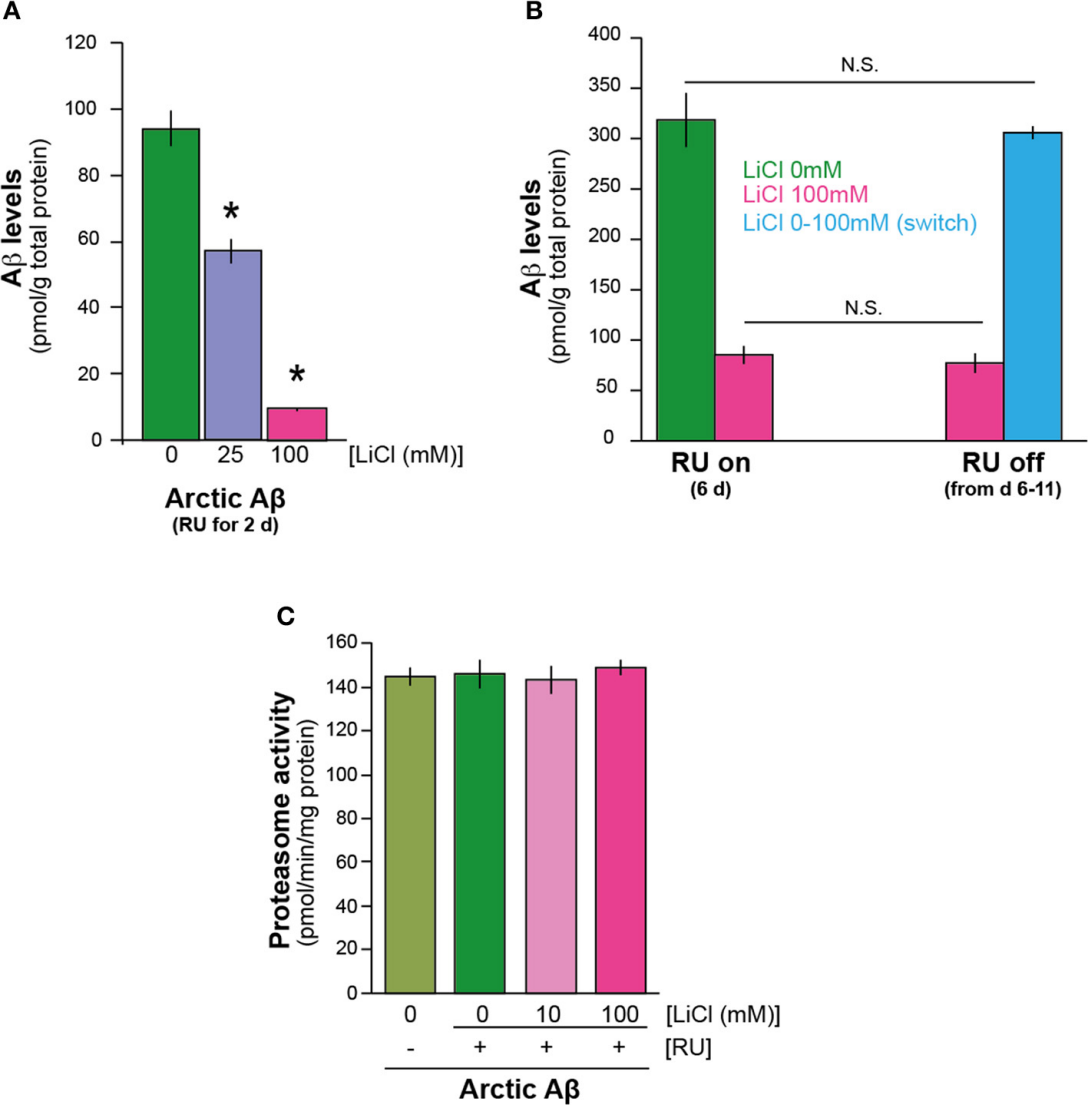
**Lithium reduced the level of Aβ42 peptide in the adult *Drosophila* nervous system**. **(A)** Lithium reduced amyloid levels of Arctic Aβ42 flies at a very early time-point. Protein levels of UAS-ArcAβ42/UAS-gfp;elavGS/+ flies on +RU486 SY medium, and UAS-ArcAβ42/UAS-gfp;elavGS/+ flies on +RU +lithium (LiCl), were measured by ELISA at 2 days post-induction (see Materials and Methods). Data were compared using One-Way ANOVA, number of independent tests (*n*) = 4. ^*^P < 0.01 and ^*^P < 0.001 when comparing UAS-ArcAβ42/UAS-gfp;elavGS/+ +RU to UAS-ArcAβ42/UAS-gfp;elavGS/+ +RU +LiCl 25 or 100 mM respectively. **(B)** Protein levels of UAS-ArcAβ42/UAS-gfp;elavGS/+ flies on RU + 100 mM LiCl medium at 6 days post induction, and then switched to –RU LiCl for 5 days were not significantly different from ArcAβ42/UAS-gfp;elavGS/+ flies on RU + 100 mM LiCl medium for 6 days, *P* = 0.6, nor were UAS-ArcAβ42/UAS-gfp;elavGS/+ flies on +RU 6 days post induction different in comparison to UAS-ArcAβ42/UAS-gfp;elavGS/+ flies on +RU –LiCl for 6 days switched on to –RU + LiCl for 5 days, *P* = 0.7. Data were compared using One-Way ANOVA, number of independent tests (*n*) = 4. **(C)** Proteasome activity, as measured using the fluorogenic peptide substrate LLVY-AMC, was not changed in flies over-expressing Arctic Aβ42 with or without LiCl treatment. Data are presented as mean activities (pmol/min/mg protein) ± s.e.m., *P* = 0.72 and 0.82 for 10 and 100 mM Li (*n* = 5). ^*^p < 0.01 and 0.001 for 25 and 100 respectively.

To understand the mechanism/s by which lithium reduces Aβ42 protein level, we first investigated the speed of Aβ42 accumulation after induction of expression, in the presence and absence of lithium. We measured total Aβ protein in the flies expressing Arctic mutant Aβ42 specifically in adult neurons (elav gene switch system was used to induce Aβ expression with the activator mifepistrone, RU486/RU) (Sofola et al., 2010) at a very early time-point, 2 days post Aβ induction. We found by ELISA analysis that even at this early time point, UAS-ArcAβ42/UAS-gfp;elavGS/+ +RU +Li flies that were fed either 25 or 100 mM lithium showed a major reduction in total Aβ burden in comparison to UAS-ArcAβ42/UAS-gfp;elavGS/+ +RU controls, *P* < 0.01 and *P* < 0.001 respectively (Figure 1A). These data suggest that lithium may affect synthesis rather than degradation of Aβ, because a reduction in Aβ levels was already present at such an early time point.

Next, we directly assessed the effects of lithium on degradation of Aβ42. We induced Aβ expression for 6 days in the presence of lithium (UAS-ArcAβ42/UAS-gfp;elavGS/+ +RU +Li), then stopped Aβ induction and divided the flies randomly into two groups, in one of which lithium treatment was continued for a further 5 days (UAS-ArcAβ42/UAS-gfp;elavGS/+ -RU+Li), while no lithium was added to the food of the control group (UAS-ArcAβ42/UAS-gfp;elavGS/+ -RU -Li). Thus, if lithium promoted degradation of Aβ, then the level of Aβ at the end of the treatment period should have been lower in the flies with continued lithium treatment. Aβ levels at the end of the 11-day period did not differ significantly between the two groups (Figure 1B), indicating that lithium treatment did not enhance degradation of Aβ. To confirm this finding, we again induced Aβ expression for 6 days, but in the absence of lithium (UAS-ArcAβ42/UAS-gfp;elavGS/+ +RU -Li), then stopped Aβ induction and divided the flies randomly into two groups, one of which was frozen immediately, while in the other, lithium treatment was administered for 5 days (UAS-ArcAβ42/UAS-gfp;elavGS/+ -RU +Li). Aβ levels at the end of the 5-day lithium treatment period did not differ significantly from levels in the untreated, 6-day old flies (Figure 1B). These data suggest that lithium does not promote Aβ degradation or clearance, and point instead to a role in Aβ protein synthesis. We also tested if lithium modulated proteasome activity in Aβ expressing flies (2 days post Aβ induction) and found that at 10 and 100 mM doses it did not (Figure 1C), consistent with our finding that lithium does not appear to affect Aβ protein degradation.

### Lithium down-regulated translation

**Figure 2:**
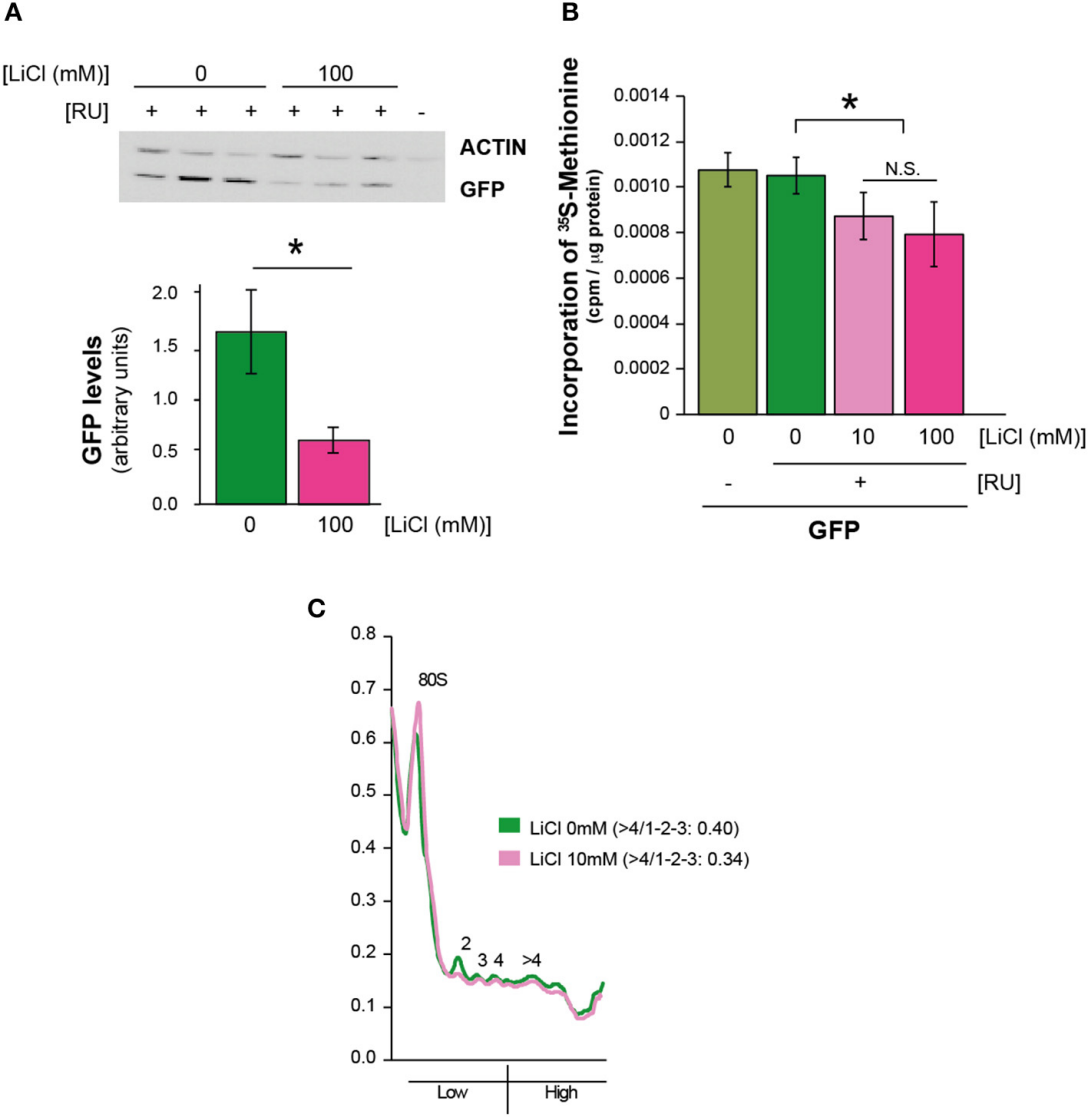
**Lithium reduced overall protein synthesis/translation**. **(A)** Protein levels of UAS-egfp/+;elavGS/+ flies on +RU486 SY medium, and UAS-egfp/+;elavGS/+ flies on +RU +100 mM LiCl were measured by Western blot analyses at 7 days post-induction (see Materials and Methods). GFP levels were significantly reduced when comparing UAS-egfp/+;elavGS/+ +RU to UAS-egfp/+;elavGS/+ +RU +LiCl 100 mM, ^*^P < 0.001. Data were compared using One-Way ANOVA, number of independent tests (*n*) = 6. **(B)** Protein synthesis was reduced in pooled UAS-egfp/+;elavGS/+ +RU +LiCl 10 mM and Li 100 mM treated flies in comparison to UAS-egfp/+;elavGS/+ +RU flies measured by ^35^S-methionine incorporation, ^*^P < 0.05. Data were analyzed by One-Way ANOVA (*n* = 7 for +RU and *n* = 13 for +RU +LiCl treated flies). **(C)** Polysome profiles showed a reduction in translation in lithium treated Aβ expressing flies, measured by calculating the area under the different fractions, and their ratios (area under the first 3 peaks for low fraction, and >4 for high, ratio >4/1-2-3) *P* < 0.001. Data were analyzed by paired *t*-test (*n* = 5). One representative figure is shown.

To determine whether the effect of lithium on Aβ protein level was specific to Aβ, we investigated whether it affected the level of green fluorescent protein (GFP), a protein that is irrelevant to AD pathology. Surprisingly, we found that 100 mM lithium significantly reduced levels of GFP in the neurons of adult flies (UAS-egfp/+;elavGS/+ +RU), *P* = 0.01 (Figure 2A). These data suggest that lithium affects protein synthesis through a mechanism that is not specific to Aβ. To determine whether overall level of translation was reduced by lithium, we carried out S-methionine radioactive tracer experiments in UAS-egfp/+;elavGS/+ flies. Interestingly, we found lower ^35^S-methionine incorporation into protein in the bodies of flies treated with lithium (pooled data of 10 and 100 mM lithium) in comparison to untreated control flies (Figure 2B), *P* < 0.05. Based on this finding, we measured the effect of lithium on polysome profiles of Aβ flies, as an indication of activity of the translation machinery. There was a significant reduction in the ratio of high (polysomes >4) to low (monosome/polysome 1-2-3) fraction in flies treated with lithium (10 mM) (UAS-ArcAβ42/+;elavGS/+ +RU +Li) in comparison to untreated controls (UAS-ArcAβ42/+;elavGS/+ +RU flies), 0.34 vs. 0.40, *P* < 0.001 (Figure 2C), again demonstrating that lithium reduced activity of the translation machinery, possibly through a stall in translation initiation.

**Figure 3:**
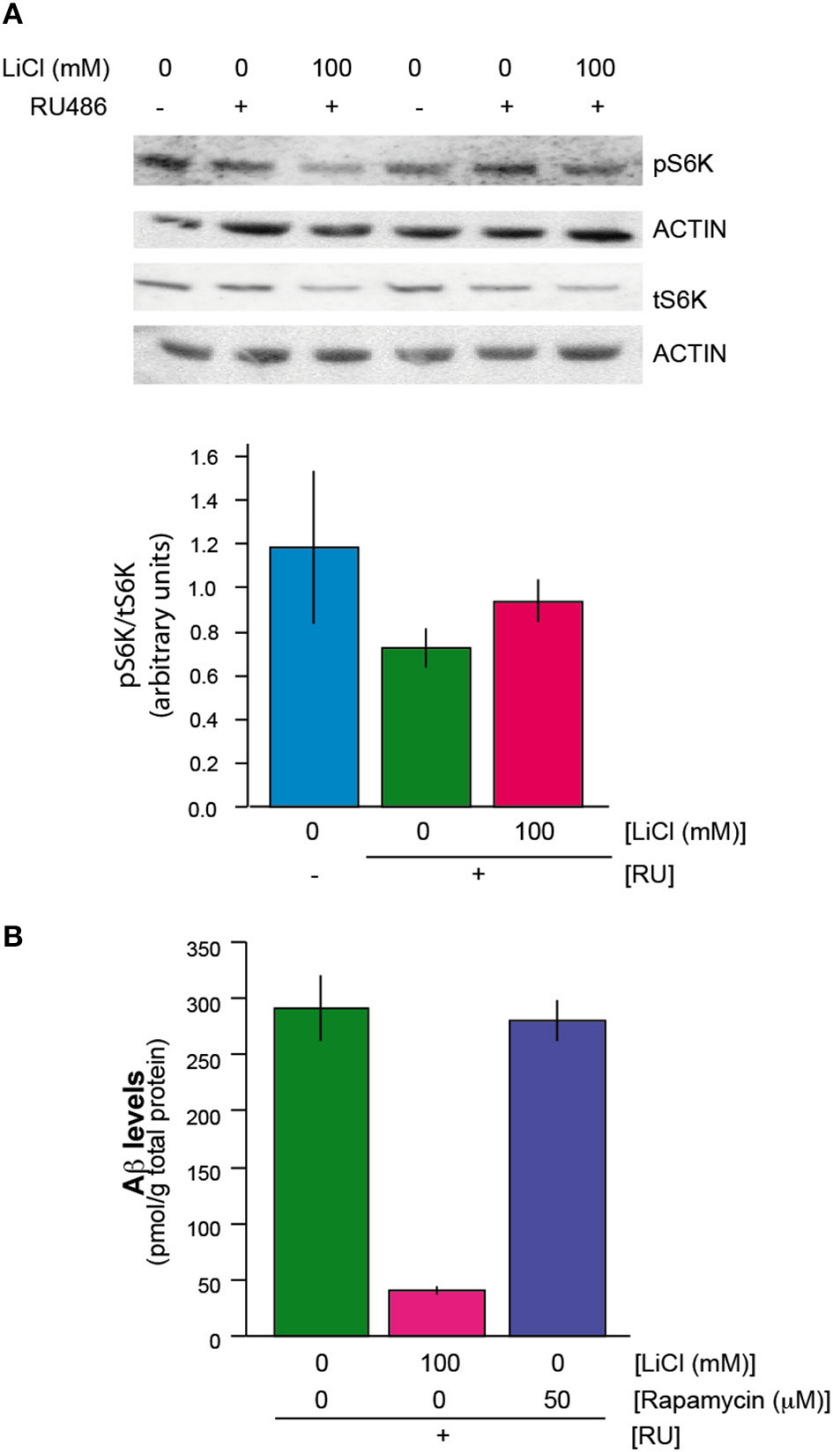
**(A)** Lithium reduced protein levels of phosphorylated and total S6K. There was no change in the ratio of phosphorylated S6K to total S6K protein levels when UAS-ArcAβ42/+;elavGS/+ +RU +LiCl 100 mM flies are compared to UAS-ArcAβ42/+;elavGS/+ +RU controls, *P* = 0.2 (*n* = 3) **(B)** Rapamycin did not reduce Aβ levels in UAS-ArcAβ42/+;elavGS/+ +RU +50 μM flies in comparison to UAS-ArcAβ42/+;elavGS/+ +RU untreated flies, *P* = 0.8 (*n* = 3).

We next determined whether lithium exerted its effect on translation through an effect on the activity of the mechanistic target of rapamyicin (mTOR) pathway, which is involved in control of translation. S6K is a phosphorylation target of mTOR kinase in the mTORC1 complex (Bjedov and Partridge, 2011); interestingly, both phosphorylated S6K and total S6K were reduced in Aβ flies treated with lithium (UAS-ArcAβ42/+;elavGS/+ +RU +Li) in comparison to untreated flies (UAS-ArcAβ42/+;elavGS/+ +RU) (Figure 3A). However, the ratio of phosphorylated S6K to total S6K was not significantly different between the two groups (Figure 3A). In these blots, because of the possibility that total protein content of the flies could have been altered by lithium, samples derived from a fixed number of fly heads were used. Interestingly, actin levels did not seem to be affected by lithium and were used as an additional loading control. This finding suggests that either the turnover rate of actin is very low, or there is a subset of proteins that is not down-regulated by lithium. Treatment of Aβ expressing flies (UAS-ArcAβ42/+;elavGS/+ +RU) with rapamycin, an inhibitor of mTOR that inhibits translation and extends lifespan in *Drosophila* (Bjedov et al., 2010) also did not reduce the levels of Aβ in the Aβ-expressing flies (Figure 3B), and nor did it ameliorate the Aβ-induced toxicity (Supplemental Figure 1A). These data suggest that lithium did not act through the mTOR pathway to reduce translation in the Aβ-expressing flies, and that the reduced levels of total and phosphorylated S6K were instead a consequence of a general reduction in translation.

**Figure 4:**
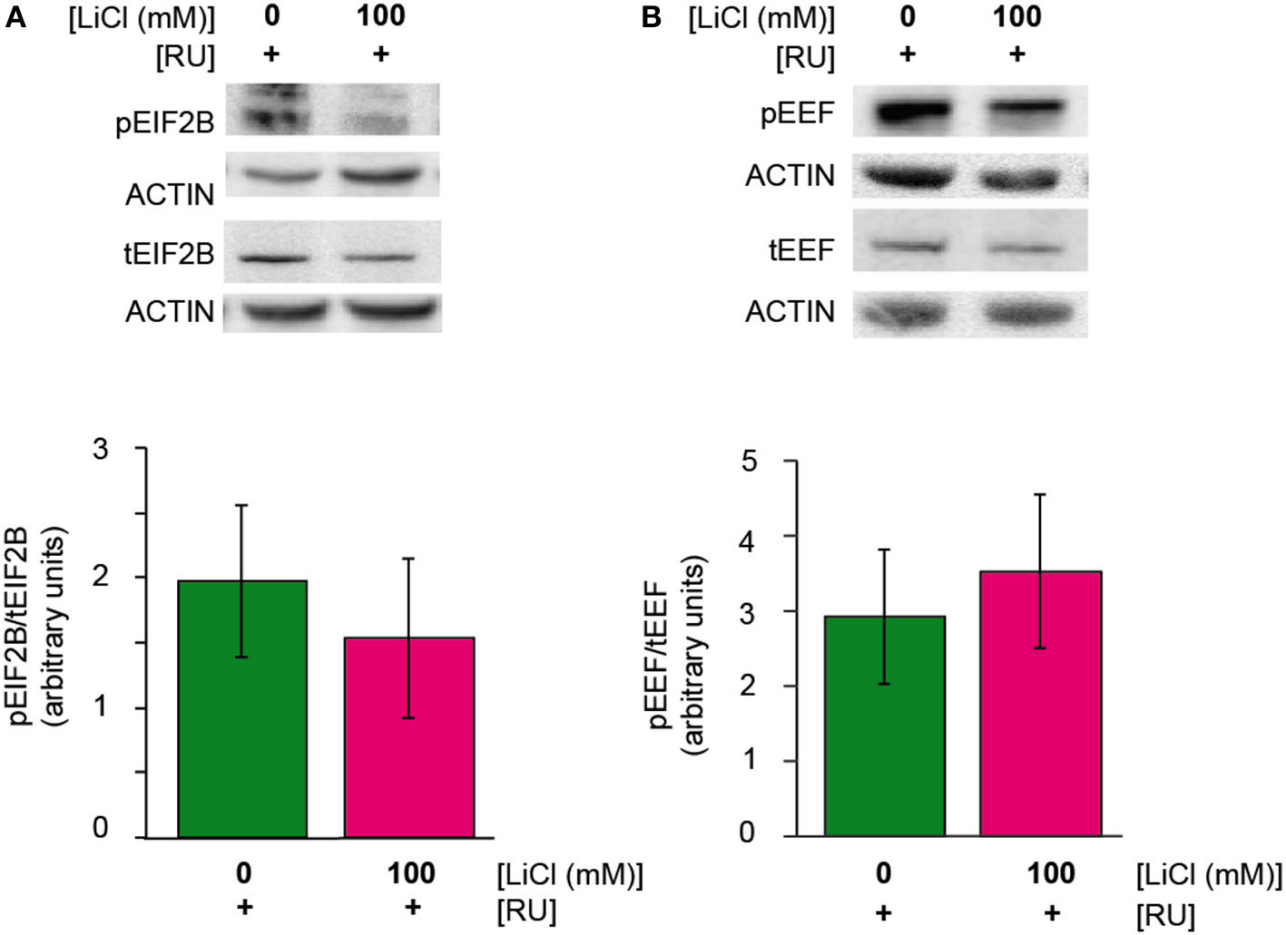
**(A,B)** Lithium reduced protein levels of phosphorylated and total eIF2Be and eEF-2. There was no change in the ratio of phosphorylated eIF2Be or eEF-2 to total protein levels of eIF2Be or eEF-2 in UAS-ArcAβ42/+;elavGS/+ +RU +LiCl 100 mM flies in comparison to UAS-ArcAβ42/+;elavGS/+ +RU controls, *P* = 0.6 and 0.7 respectively (*n* = 3).

Lithium has been shown to regulate the translation initiation factor eIF2B in rats (Bosetti et al., 2002) and the elongation factor, eEF-2 in SH-SY5Y cells and mice (Karyo et al., 2010). We investigated whether lithium reduced protein synthesis by regulating one or both of these factors. Again, we observed a reduction in both the phosphorylated and total forms of eIF2B-epsilon and eEF-2 in Aβ expressing flies treated with 100 mM lithium (UAS-ArcAβ42/+;elavGS/+ +RU +Li) in comparison to untreated flies (UAS-ArcAβ42/+;elavGS/+ +RU), but the ratio of phosphorylated to total eIF2B-epsilon or eEF-2 was not significantly affected (Figures 4A,B). These data again demonstrate that lithium down-regulated protein levels, but they do not point to a specific role for eIF2B-epsilon or eEF-2.

Indeed, a clear-cut explanation of the effects of lithium may be elusive, since translation of many proteins involved in the protein synthesis machinery is presumably inhibited by lithium. Furthermore, we used a high dose of lithium (100 mM) to maximize our chances of identifying these downstream factors that are responsible for the effect of lithium on translation, and were unable to identify a specific role for the proteins we tested; and thus did not test lower concentrations of lithium. Overall, the data suggest that the reduction in Aβ levels we observe upon lithium treatment is as a consequence of reduction in protein synthesis, probably because of reduced expression of multiple proteins involved in initiation of translation, but do not identify the specific targets responsible.

### Lithium also inhibits protein synthesis in fission yeast

**Figure 5:**
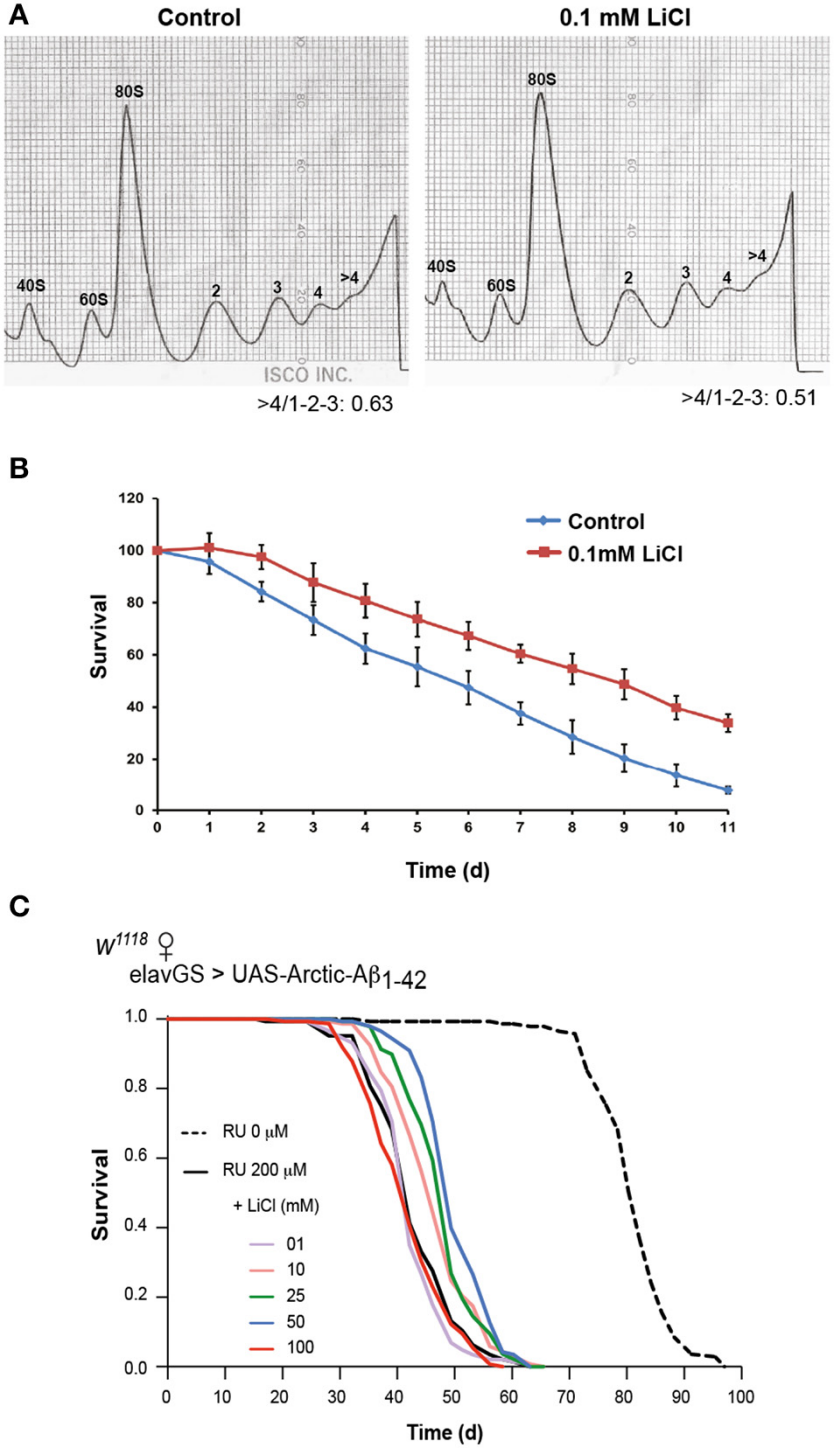
**Lithium reduced overall protein synthesis/translation and extended lifespan in fission yeast**. **(A)** Polysome profiles showed a reduction in translation in lithium treated fission yeast cells. The area under the different fractions and their ratios were calculated (area under the first 3 peaks for low fraction, and >4 for high, fraction, ratio >4/1-2-3). Data were analyzed by paired *t*-test (*n* = 3), *P* < 0.001. **(B)** 0.1 mM LiCl increased the chronological median lifespan of fission yeast (paired *t*-test comparing triplicates of lifespan values at 50% survival/medial lifespan, *P* = 0.001). **(C)** Survival curves of flies expressing Arctic Aβ42 with or without lithium treatment are depicted, data were compared using the log-rank test, comparing UAS-ArcAβ42/+;elavGS/+ +RU +LiCl 1, 10, 25, 50 or 100 mM flies to UAS-ArcAβ42/+;elavGS/+ +RU controls. No significant difference was seen between UAS-ArcAβ42/+;elavGS/+ +RU controls and UAS-ArcAβ42/+;elavGS/+ +RU +LiCl 1 or 100 mM, *P* = 0.24 and 0.17 respectively, a significant difference was observed with 10, 25, and 50 mM LiCl, *P* < 0.001.

Reduction in protein synthesis has been frequently linked to increased lifespan, possibly attributable to both a reduction in energy consumption, because translation requires a significant amount of the energy budget, and a reduction in mis-translated polypeptides (Browne and Proud, 2002; Proud, 2002; Hansen et al., 2007; Hipkiss, 2007). Additionally, lithium has been shown to cause significant lifespan-extension in the nematode worm *C. elegans* and in *Drosophila* (McColl et al., 2008; Kasuya et al., 2009; Zarse et al., 2011). Therefore, we decided to turn into a simpler system to determine whether lithium has an evolutionarily conserved effect on translation, and whether it has an associated effect on lifespan. Unicellular yeasts have been pivotal in the advancement of understanding of mechanisms of ageing (Kaeberlein et al., 2005; Rallis et al., 2013). Fast-growing fission yeast cells in mid-log phase were treated with 0.1 mM lithium chloride for 1 h and their translational profiles were compared to those of untreated (control) cells. Following lithium treatment, translation was reduced: high (polysomes >4) to low (monosome/polysome 1-2-3) ratios were lower (0.51) in lithium treated yeast cells in comparison to control cells (0.63), *P* < 0.001 (Figure 5A). Interestingly, the same dose of lithium extended the chronological lifespan of fission yeast, *P* = 0.001 (Figure 5B). Yeast is hence an ideal model organism in which to perform a genetic screen to identify the relevant targets of lithium for reduced protein synthesis and increased lifespan.

### Lithium extended lifespan of flies expressing Aβ

Since we observed an association between reduced global protein synthesis and increased longevity in fission yeast, we determined whether lithium could also extend the survival of Aβ expressing flies. We measured lifespan of flies expressing Arctic Aβ42 (UAS-ArcAβ42/+;elavGS/+ +RU) and treated chronically with different concentrations of lithium from two days post-eclosion. Flies expressing Arctic Aβ42 in adult neurons had a shortened median and maximum lifespan as previously reported (Sofola et al., 2010), which could be extended with 10, 25, and 50 mM (*P* < 0.001 for all doses), but not with 1 or 100 mM lithium (Figure 5C). The data suggest that above a certain a threshold, somewhere between 50 and 100 mM, lithium no longer rescued the shortened lifespan of flies expressing Aβ, possibly because it became toxic. However, this toxicity with a high lithium dose was only evident when taken long term and/or in older flies, since both 10 mM (Supplemental Figure 1B) and 100 mM lithium previously published (Sofola et al., 2010) were able to protect against the Aβ-induced locomotor dysfunction measured after shorter term treatment and earlier in life.

## Discussion

Human life expectancy continues to increase at a steady rate in most countries worldwide, and has done so by almost 3 months per year in the last 160 years (Oeppen and Vaupel, 2002). Therefore, it is of great importance to tackle ageing-related diseases such as AD, because they are becoming increasingly prevalent. Because age is the biggest risk factor for AD, interventions that promote general increases in health during ageing could also be important and beneficial in AD.

Lithium is becoming increasingly implicated as a drug that can ameliorate ageing and neurodegenerative diseases. Several groups have shown that it extends lifespan in model organisms such as the nematode worm *C*. *elegans* and *Drosophila* (McColl et al., 2008; Kasuya et al., 2009; Zarse et al., 2011). Here, we showed that it also extends lifespan in fission yeast *Schizosaccharomyces pombe*, highlighting that this effect is conserved over large evolutionary distances. Fission yeast is an ideal organism for genetic screens, and future work should identify the molecular targets of lithium both for control of protein synthesis and of lifespan. Furthermore, slightly higher levels of lithium present in the drinking water have been reported as associated with reduced mortality in a Japanese human population (Zarse et al., 2011).

A substantial body of work has demonstrated that several neurodegenerative diseases and neurological disorders, including but not confined to stroke, schizophrenia, Fragile X syndrome, Huntington's disease and Parkinson's disease, benefit from the therapeutic properties of lithium (Chiu et al., 2011). In addition, several studies have investigated whether lithium has a beneficial effect in AD pathogenesis. Clinical trials conducted with lithium have yielded conflicting results; some have found benefits, whilst others have not (Nunes et al., 2007; Macdonald and Briggs, 2008; Hampel et al., 2009). Interestingly, a correlative study conducted in patients with bipolar disorder, suggested that patients that had been on chronic lithium treatment showed a reduced incidence of AD in comparison to patients that had not been on treatment (Nunes et al., 2007). And a more recent small-scale clinical trial on mild cognitive impaired (MCI) patients found that low doses of lithium slowed cognitive decline (Forlenza et al., 2011). The investigators suggested that a reason for the previous conflicting data on the efficacy of lithium was probably attributable to the pathological states/stages at which the patients were given lithium. It is becoming increasingly evident that drug trials are most likely to yield positive effects when initiated early, at the MCI stage.

Our results add to existing data suggesting that lithium could be beneficial in ameliorating Aβ toxicity, and should be considered for a potential large-scale trial on MCI patients. It has the added advantage of being an already approved drug, used to treat bipolar patients. It does have side-effects, but these are minimal at the low doses used in the recent small-scale clinical study (Forlenza et al., 2011). We also found that there are limits to the beneficial/therapeutic benefits of lithium in fission yeast in chronological lifespan—lithium was unable to increase chronological lifespan at higher doses (data not shown) as well as in the *Drosophila* AD model. Previously, we showed in Sofola et al. (2010) that administering both 30 and 100 mM lithium into the fly food are effective in modulating Aβ neuronal toxicity as evidenced by the improved locomotor function in young flies (Sofola et al., 2010). These lithium concentrations were initially chosen based on the paper by Dokucu et al. (2005)—they showed that lithium concentrations ranging from 10 to 100 mM lithium in the fly food translates to roughly 0.05–0.4 mM in the fly tissue (Dokucu et al., 2005), so well below the toxic levels in patients and mice (Wood et al., 1986; Schou, 2001; Can et al., 2011). In this paper, we show that both 25 and 100 mM lithium reduce Aβ levels in a dose dependent manner at an early time point. We also find that lower doses of Lithium (10 and 25 mM) rescue the shortened longevity of the Aβ flies, but 100 mM lithium was unable to extend lifespan when given to the flies throughout adulthood. It will be important to determine the therapeutic thresholds for lithium in patients that could offer therapeutic benefits without overt side effects.

Similar to the published data on the role of GSK-3 inhibition in down-regulating translation in HCC1806 cells (Shin et al., 2014), we find that lithium is able to reduce translation in fission yeast and flies, suggesting that perhaps some of the effect of lithium on translation down-regulation is via GSK-3 inhibition. However, this is correlative and future work will involve carrying out epistasis interactions between lithium and GSK-3, and identifying molecular targets of GSK-3 and lithium for control of protein synthesis.

Nonetheless, our study highlights the potential benefits of lithium through down-regulation of translation, associated with extension of lifespan in very distantly related organisms. By reducing protein synthesis, lithium may reduce the increased proteostatic burden in ageing, a recognized hallmark of ageing (López-Otín et al., 2013). Lithium is also of specific benefit in AD, because of its ability to down-regulate translation, and hence levels of proteins involved in promoting the presence of toxic Aβ.

The mutant Arctic Aβ42 protein present in the transgenic flies used in this study has a propensity to aggregate faster than wild type Aβ (Spires and Hyman, 2005). However, we have observed both soluble and insoluble Aβ in the Arctic Aβ42 flies [(Rogers et al., 2012), and data not shown]; and the ability of lithium to reduce translation of the Aβ peptide without affecting its clearance may lower the level of soluble Aβ. In a wider context, lithium might be beneficial in ameliorating toxicity of AD by lowering expression of APP and of proteins that are involved in the generation of Aβ from APP. Our model does not express full length APP, and may therefore not include other potential/additional benefits of lithium on Aβ toxicity. As well as the increased ratio of Aβ42 to Aβ 40 peptide observed in familial AD cases with APP mutations (De Jonghe et al., 2001), increased levels of APP could also contribute to AD pathogenesis. Indeed, patients with Down syndrome have a high risk of developing AD possibly due to trisomy of the APP gene which leads to increased APP expression (Wiseman et al., 2009). Also, several mutations in the APP promoter region were found to significantly increase APP expression in SH-SY5Y cells, and were associated with risk for AD (Theuns et al., 2006).

The ability of lithium to down-regulate translation could therefore be beneficial at several stages in AD pathogenesis. Lithium might also have therapeutic benefits for other neurodegenerative disorders that are caused by over-expression of wild type or mutant forms of proteins such as α-synuclein in Parkinson's disease. Lithium could also reduce the production of mis-translated polypeptides, and free proteases or/and chaperones that can then participate in cellular proteostasis (Proud, 2002; Singleton et al., 2003). Furthermore, diseases where protein turnover is compromised by loss of function of the degradation machinery could also benefit from lowering the burden of protein production hence reducing cellular stress. This could be particularly important in lysosomal storage diseases, where the intrinsic function of the degradative machinery is compromised (Kinghorn, 2011; Sarkar et al., 2013). Moreover, induction of autophagy in some cases increases the load of an already dysfunctional lysosome, worsening the cellular proteostatic stress (Wong and Cuervo, 2010; Nixon and Yang, 2011). Hence lowering the production of proteins could again be a viable mechanism to restore proteostasis. Other neurodegenerative models where the role of lithium in lowering protein translation could be beneficial are, for example, the *Drosophila* models of Pink1 and Parkin, which do not include the over-expression of toxic proteins (Whitworth et al., 2006; Castillo-Quan, 2011). Flies lacking either of these proteins accumulate unfolded proteins in mitochondria, resulting in mitochondria impairment (Pimenta de Castro et al., 2012). It would be interesting to study whether lithium could ameliorate mitochondrial stress by reducing the production of the proteins accumulating in the mitochondria of the Pink1 or parkin null flies. Lithium could hence be a useful drug with an overall benefit for health during ageing and protection against AD and other neurodegenerative diseases.

### Conflict of interest statement

The authors declare that the research was conducted in the absence of any commercial or financial relationships that could be construed as a potential conflict of interest.

## Supplementary material

Supplemental Figure 1 **(A)** Rapamycin did not rescue the climbing phenotype of UAS-ArcAβ42/+;elavGS/+ flies on +RU486 +200 μM rapamycin, *P* = 0.83 **(B)** Lithium suppressed the locomotor dysfunction phenotype, *P* < 0.05. Climbing ability of UAS-ArcAβ42/UAS-gfp;elavGS/+ +RU486 and UAS-ArcAβ42/+;elavGS/+ flies on +RU486 +LiCl 10 mM SY medium or +Rapamycin 200 μM was assessed at the indicated time-points (see Materials and Methods). Data are presented as the percentage climbing performance of flies ± s.e.m. [Two-Way ANOVA, number of independent tests (*n*) = 3].
