# Supplementary figures and images for: Lithium suppresses Aβ pathology by inhibiting translation in an adult Drosophila model of Alzheimer's disease
Source: Front Aging Neurosci. 2014 Jul 30;6:190. doi: 10.3389/fnagi.2014.00190 (PMC4115666; doi:10.3389/fnagi.2014.00190)

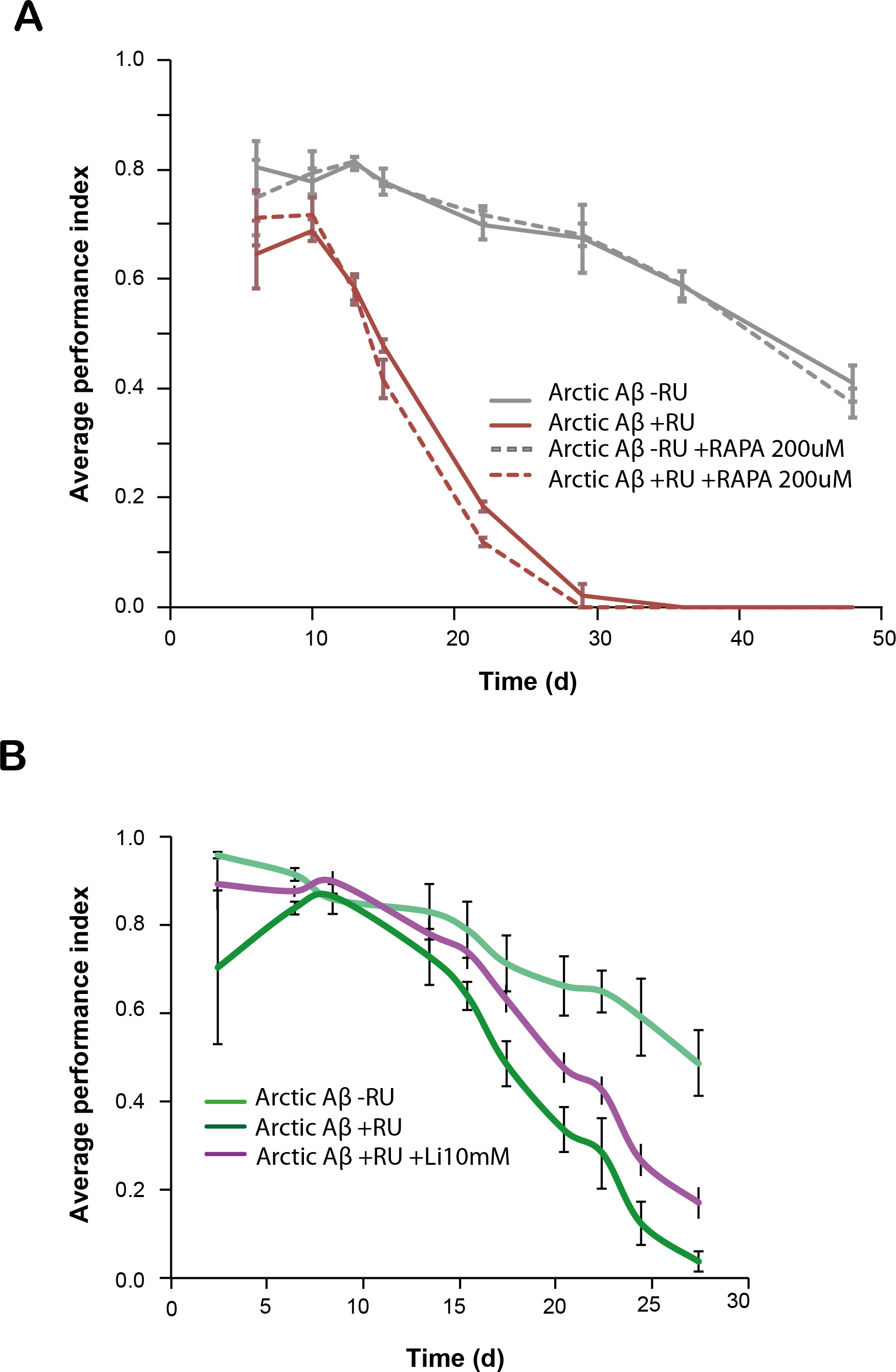

Supplement: Supplemental Figure 1 — (A) Rapamycin did not rescue the climbing phenotype of UAS-ArcAβ42/+;elavGS/+ flies on +RU486 +200 μM rapamycin, P = 0.83 (B) Lithium suppressed the locomotor dysfunction phenotype, P < 0.05. Climbing ability of UAS-ArcAβ42/UAS-gfp;elavGS/+ +RU486 and UAS-ArcAβ42/+;elavGS/+ flies on +RU486 +LiCl 10 mM SY medium or +Rapamycin 200 μM was assessed at the indicated time-points (see Materials and Methods). Data are presented as the percentage climbing performance of flies ± s.e.m. [Two-Way ANOVA, number of independent tests (n) = 3]. [file Image1.JPEG]
